# IL-6 Blockade Enhances the Efficacy of CDK4/6 Inhibitor in *BRCA1*-Mutant Triple-Negative Breast Cancer Cells

**DOI:** 10.3390/cells14201602

**Published:** 2025-10-15

**Authors:** Li Pan, Changyou Shi, Joungil Choi, Jiayuh Lin

**Affiliations:** 1 Department of Biochemistry and Molecular Biology, University of Maryland School of Medicine, Baltimore, MD 21201, USA; li.pan@som.umaryland.edu (L.P.); changyou.shi@som.umaryland.edu (C.S.); 2 Flow Cytometry Core Service, VA Maryland Healthcare System, 10N. Greene St., Baltimore, MD 21201, USA; joungil.choi@va.gov

**Keywords:** triple-negative breast cancer (TNBC), *BRCA1* mutation, CDK4/6, abemaciclib, IL-6 inhibitor, bazedoxifene, IL-6/STAT3 signaling

## Abstract

Breast cancer gene 1 (*BRCA1*) is a tumor suppressor gene essential for DNA repair, and its mutations are linked to aggressive breast cancers with poor prognosis. While poly (ADP-ribose) polymerase (PARP) inhibitors benefit some patients with *BRCA1*-mutant, human epidermal growth factor receptor 2 (HER2)-negative metastatic breast cancer, issues such as limited efficacy and drug resistance persist. This is especially critical for triple-negative breast cancer (TNBC), which lacks targeted therapies. Cyclin-dependent kinase 4/6 (CDK4/6) inhibitors such as abemaciclib—FDA-approved for estrogen receptor (ER)-positive/HER2-negative breast cancer—are emerging as potential treatments for TNBC. We evaluated abemaciclib in *BRCA1*-mutant TNBC cell lines (SUM149, HCC1937, and MDA-MB-436) and found them to be sensitive to the drug. However, treatment induced cellular senescence and Interleukin-6 (IL-6) secretion, which may promote drug resistance. To address this, we inhibited IL-6 signaling using bazedoxifene or glycoprotein 130 (GP130) siRNA, and both of which enhanced abemaciclib sensitivity. Combination treatment with bazedoxifene and abemaciclib synergistically inhibited cell migration and invasion, and induced apoptosis. In a mammary fat pad TNBC tumor model, the combination treatment significantly suppressed SUM149 tumor growth more than either agent alone. These findings support co-targeting IL-6 and CDK4/6 as a novel therapeutic strategy for *BRCA1*-mutant TNBC.

## 1. Introduction

Triple-negative breast cancer (TNBC) is the most aggressive subtype of breast cancer, characterized by the absence of estrogen receptor (ER), progesterone receptor (PR), and human epidermal growth factor receptor 2 (HER2) [1]. Among TNBC cases, a subset harbors breast cancer gene 1 (*BRCA1*) mutations, which impair DNA repair through homologous recombination (HR) deficiency and make these tumors more sensitive to DNA-damaging agents [2,3]. *BRCA1*-mutated TNBC often exhibit more aggressive clinical features than their *BRCA1* wild-type counterparts, including elevated histologic grade, pronounced genomic instability, and an increased likelihood of metastasis [4,5]. Although *BRCA1*-mutated TNBC tends to display aggressive clinical behavior, its deficiency in HR repair renders it particularly vulnerable to DNA-damaging treatments, such as platinum-based chemotherapies (e.g., cisplatin, carboplatin) and poly (ADP-ribose) polymerase (PARP) inhibitors (e.g., olaparib, talazoparib) [6,7]. While PARP inhibitors have shown clinical benefit, resistance remains a significant challenge, limiting their long-term efficacy [8]. Additionally, the lack of targeted therapies for TNBC, coupled with its highly metastatic nature and poor prognosis, underscores the need for novel therapeutic strategies to improve outcomes for patients with *BRCA1*-mutant TNBC.

Cyclin-dependent kinases 4 and 6 (CDK4/6) are functionally related enzymes essential for controlling cell cycle progression [9]. Upon forming a complex with cyclin D, CDK4/6 initiates phosphorylation of the retinoblastoma (Rb) protein, leading to the dissociation of E2 promoter-binding factor (E2F) transcription factors [10]. This cascade facilitates the transition from the G1 to S phase, driving DNA synthesis and cell proliferation [11]. In numerous cancers, particularly hormone receptor-positive breast cancer, hyperactivation of the CDK4/6–cyclin D–Rb signaling axis drives unchecked cell proliferation [12,13]. As a result, CDK4/6 inhibitors hinder cell cycle progression by preventing Rb phosphorylation, thereby halting cells in the G1 phase and suppressing tumor development and expansion [14]. At present, combining CDK4/6 inhibitors (such as palbociclib, ribociclib, or abemaciclib) with endocrine therapy represents the leading treatment strategy for individuals with hormone receptor positive and HER2 negative advanced breast cancer [15,16]. Their clinical utility in TNBC remains under investigation, with emerging evidence suggesting potential benefit particularly in specific molecular subtypes, such as the luminal androgen receptor (LAR) subtype [17]. However, acquired resistance remains a significant clinical challenge. Various mechanisms of resistance to CDK4/6 inhibitors have been identified, including the interleukin-6 (IL-6)/signal transducer and activator of transcription 3 (STAT3) pathway [18], loss of functional Rb [19], increased activity of cyclin E1 and Cyclin-dependent kinases 2 (CDK2) [20], activation of the phosphoinositide 3-kinase (PI3K)/protein kinase B (PKB, also known as AKT)/mechanistic target of rapamycin (mTOR) and mitogen-activated protein kinase (MAPK) pathways [21], and compensatory signaling via the cyclin D1/CDK2 axis [22].

Notably, increasing evidence highlights aberrant activation of the IL-6/STAT3 signaling pathway as a key contributor to tumor growth and resistance to therapy in cancer [23]. IL-6-mediated activation of STAT3 promotes transcription of genes involved in cell survival, proliferation, and cell cycle progression, effectively bypassing CDK4/6 inhibition [24,25,26]. This pathway not only supports tumor growth despite CDK4/6 blockade but also contributes to a more aggressive and therapy-resistant phenotype, underscoring the therapeutic potential of dual inhibition strategies targeting both CDK4/6 and IL-6/STAT3 in TNBC. Our lab has confirmed that CDK4/6 inhibitor abemaciclib in combination with bazedoxifene showed synergistic inhibition in *BRCA1* proficient TNBC cells in vitro [27].

Building on this finding, the current study seeks to determine whether this two-drug combination can also demonstrate efficacy in *BRCA1*-mutant TNBCs, thereby addressing the critical challenge of PARP inhibitor resistance and extending the therapeutic potential of this strategy. In this study, we evaluated the effect of a CDK4/6 inhibitor in *BRCA1*-mutant TNBC cells and found that these cells were sensitive to abemaciclib. Treatment with abemaciclib induced cellular senescence and promoted the secretion of IL-6. Co-targeting CDK4/6 and the IL-6/STAT3 signaling pathway further suppressed cell viability, migration, and invasion, while also promoting apoptosis in *BRCA1*-mutant TNBC cells. The combination of bazedoxifene and abemaciclib enhanced antitumor activity in vivo. These findings suggest that dual inhibition of IL-6 and CDK4/6 represents a novel therapeutic approach for *BRCA1*-mutant TNBC.

## 2. Materials and Methods

### 2.1. Cell Lines and Drug Compounds

The human *BRCA1*-mutant TNBC cell line MDA-MB-436 was purchased from the American Type Culture Collection (ATCC, Manassas, VA, USA; cat. no. HTB-13), SUM149 and HCC1937 were obtained from the University of Maryland Greenebaum Comprehensive Cancer Center Translational Laboratory (Baltimore, MD, USA). The cells were cultured in Roswell Park Memorial Institute (RPMI) 1640 medium (Gibco, Thermo Fisher Scientific, Waltham, MA, USA; cat. no. 11875-093) supplemented with 10% Fetal Bovine Serum (FBS; Gibco, Thermo Fisher Scientific, cat. no. 15140-122), 5% penicillin/streptomycin (Gibco, Thermo Fisher Scientific, cat. no. 15140-122) and maintained at 37 °C in a 5% CO_2_ humidified incubator.

Bazedoxifene acetate (cat. no. 102233) and abemaciclib mesylate (cat. no. 206973) were purchased from MedKoo Biosciences, Inc. (Durham, NC, USA). Abemaciclib mesylate and bazedoxifene acetate were reconstituted in dimethylsulfoxide (DMSO; Sigma-Aldrich, St. Louis, MO, USA; cat. no. D4540) to make 20 mM stock solutions for in vitro assays. The doses of abemaciclib and bazedoxifene used for in vitro experiments were indicated in the figures.

### 2.2. Cell Viability and Proliferation Assay

The 3-(4,5-dimethylthiazol-2-yl)-2,5-diphenyltetrazolium bromide (MTT) assay was used to measure cell viability. To calculate half-maximal inhibitory concentration (IC50), *BRCA1*-mutant TNBC cells were seeded into 96-well plates at a density of 3000 cells/well in 100 µL culture medium and cultured overnight. Abemaciclib was added to cells at multiple concentrations and cultured for 72 h. After the treatment period, 20 µL of the 12 mM MTT solution (Sigma-Aldrich, St. Louis, MO, USA) was added into each well and incubated for 4 h at 37 °C. Then, 100 µL of the N, N-dimethylformamide solution (Sigma-Aldrich, St. Louis, MO, USA) was added into each well and incubated overnight at room temperature protected from light. An EL808 Ultra Microplate Reader (BioTek, Winooski, VT, USA) was used to read the absorbance at 595 nm.

Cell proliferation was detected using bromodeoxyuridine (BrdU) Cell Proliferation Assay Kit (Cell Signaling Technology, Danvers, MA, USA) according to the manufacturer’s instructions. Briefly, cells were seeded into 96-well plates at a density of 3000 cells/well and treated with abemaciclib for 72 h. After treatment, cells were incubated with 1 × BrdU solution and incubated for 2 h at 37 °C. After incubation, cells were fixed and incubated with BrdU detection antibody, followed by anti-mouse IgG and horseradish peroxidase (HRP)-linked antibody. HRP substrate 3,3′,5,5′-Tetramethylbenzidine (TMB) was used to develop color, and the absorbance at 450 nm was read using an EL808 Ultra Microplate Reader (BioTek, Winooski, VT, USA).

### 2.3. Drug Combination Analysis

Cells were seeded into 96-well plates at a density of 6000 cells/well and treated with bazedoxifene alone, abemaciclib alone and their combination for 24 h. MTT data was analyzed to determine the Combination Index (CI) using CompuSyn software 1.0. The CI values defined synergistic (CI < 1), additive (CI = 1), and antagonistic (CI > 1) effects.

### 2.4. Senescence Associated Beta-Galactosidase (SA-β-gal) Staining

Cells were treated with 2.5 µM abemaciclib for 72 h followed by washing with phosphate buffered saline (PBS; Quality Biological, Gaithersburg, MD, USA; cat. no. 114-058-101) and fixing with 4% paraformaldehyde for 10 min at room temperature. After washing with PBS twice, cells were incubated with the staining buffer at 37 °C overnight and protected from light. The staining buffer contains 150 mM sodium chloride, 2 mM magnesium chloride, 5 mM potassium ferricyanide, 5 mM potassium ferrocyanide, and 1 mg/mL X-Gal in 40 mM citric acid/sodium phosphate solution (pH 6.0). After incubation, cells were washed with PBS and rinsed with 50% dimethyl formamide for 2 min to dissolve the precipitated crystals. Images were acquired on an Olympus IX83 inverted bright-field microscope (Olympus, Tokyo, Japan) using a 20× objective.

### 2.5. Enzyme-Linked Immunosorbent Assay (ELISA)

Cells were seeded into 24-well plates at a density of 20,000 cells/well and treated with 2.5 µM abemaciclib for 72 h. The culture medium was collected and the IL-6 level was assessed using Human IL-6 Quantikine ELISA Kit (R&D Systems, Inc. Minneapolis, MN, USA) according to the manufacturer’s instructions. The results were normalized to cell numbers.

### 2.6. siRNA Transfection

Human glycoprotein 130 (GP130) siRNA and control siRNA were purchased from Santa Cruz Biotechnology (Dallas, TX, USA). Cells were seeded into 96-well plates at a density of 3000 cells/well and transfected with human GP130 siRNA and control siRNA using lipofectamine™ RNAiMAX transfection reagent (Invitrogen, Waltham, MA, USA) according to the manufacturer’s instructions. After 48 h transfection, cells were treated with abemaciclib for an additional 72 h and we proceeded with an MTT assay.

### 2.7. Cell Migration and Invasion Assay

The wound healing assay and transwell cell invasion assay were used to study cell migration and invasion. For the wound healing assay, cells were seeded into 6-well plates and cultured overnight to form confluent monolayers. A scratch was created using a 200 μL pipette tip in each well. After rinsing with PBS, cells were treated with bazedoxifene alone, abemaciclib alone, and the combination for 24 h in fresh culture medium. The initial and 24 h wound pictures were acquired, and the areas of the wounds were analyzed using ImageJ software (Version 1.54d; National Institutes of Health, Bethesda, MD, USA). For the transwell cell invasion assay, the 24-well inserts (Celltreat Scientific Products, Pepperell, MA, USA) were coated with 40 μL of 1 mg/mL Matrigel (Corning, NY, USA) at 37 °C for 2 h. Then, 500 μL culture medium containing 10% FBS was added to the well below the 24-well insert. Twenty thousand cells in 200 μL serum free medium were seeded into the inserts and treated with bazedoxifene alone, abemaciclib alone, and the combination for 24 h. Non-invading cells on the apical side of the inserts were removed using a cotton swab, and the invaded cells on the basal side of the inserts were fixed with cold methanol and stained with 0.1% crystal violet. The invaded cells were imaged and counted using ImageJ software (Version 1.54d; National Institutes of Health, Bethesda, MD, USA).

### 2.8. Cell Apoptosis Assay

Cell apoptosis was investigated by flow cytometry using APC Annexin V Apoptosis Detection Kit (Biolegend, San Diego, CA, USA) according to the manufacturer’s instructions. Briefly, cells were treated with bazedoxifene, abemaciclib, or the combination overnight. After treatment, cells were collected, washed twice with cold cell staining buffer and resuspended in Annexin V binding buffer at ~1 × 10^6^ cells/mL. Then, 5 μL Annexin V-APC and 5 μL propidium iodide (PI) were added to 100 μL of the cell suspension, mixed gently, and incubated for 15 min at room temperature in the dark. Cells were then diluted with 400 μL binding buffer and analyzed immediately using an Amnis^®^ imaging flow cytometer (Cytek Biosciences, Fremont, CA, USA) to quantify live (Annexin V^−^/PI^−^), early apoptotic (Annexin V^+^/PI^−^), and late apoptotic/necrotic cells (Annexin V^+^/PI^+^).

### 2.9. Western Blot Analysis

Cells were lysed using cell lysis buffer (Cell Signaling Technology, Danvers, MA, USA) on ice for 30 min. The concentration of the supernatant was measured using Pierce BCA Protein Assay Kit (Thermo Fisher Scientific, Waltham, MA, USA) according to the manufacturer’s instructions. The same amount of protein with 1X loading buffer was loaded into SDS-PAGE gel and transferred to a polyvinylidene difluoride membrane. After blocking in 5% non-fat milk, the blots were incubated with primary antibodies at 4 °C overnight. The primary antibodies, including rabbit anti-human phosphorylated STAT3 (P-STAT3) at Y705 (cat. no. 9145), STAT3 (cat. no. 4904), GP130 (cat. no. 3732), cyclin D1 (cat. no. 2922), survivin (cat. no. 2808), cleaved caspase3 (cat. no. 9661), and GAPDH (cat. no. 5174), were purchased from Cell Signaling Technology (Danvers, MA, USA). The next day, the blots were washed and incubated with HRP-conjugated anti-rabbit secondary antibody (Cell Signaling Technology, cat. no. 7074) in the dark for 1 h at room temperature. The signals were developed using SuperSignal^TM^ West Femto Maximum Sensitivity Substrate (Thermo Fisher Scientific, Waltham, MA, USA) and read by an Amersham Imager 680 (GE Healthcare Life Sciences, Marlborough, MA, USA).

### 2.10. Mouse Xenograft Model

Six-week-old female athymic nude mice (The Jackson Laboratory, Bar Harbor, ME, USA) were randomly assigned to four groups. SUM149 cells were selected for the in vivo studies because their growth rate allowed us to generate the large number of cells required for implantation (10 million cells per mouse, ~200 million cells in total for the study). A total of 5 × 10^6^ SUM149 cells suspended in 50 μL of matrigel (BD Biosciences, Franklin Lakes, NJ, USA) were injected bilaterally into the fourth mammary fat pads. Once tumors reached approximately 50 mm^3^ in volume, the mice (n = 3 per group, six tumors per group) received daily oral gavage treatment for 30 days with either DMSO (vehicle), bazedoxifene alone (8.8 mg/kg), abemaciclib alone (50 mg/kg), or the combination of both drugs. Tumor size was measured by length and width using a caliper ruler every two days, and tumor volume was calculated using the formula: 0.52 × L × W^2^. Body weight was also recorded every two days. At the end of the experiment, the tumors were removed and weighed. Part of the tumor tissue was lysed and analyzed by western blot for expression of P-STAT3 (Y705) and total STAT3.

### 2.11. Statistical Analysis

One-way analysis of variance (ANOVA) or Student’s *t*-test was used to compare between treatment groups. Statistical analyses were performed using GraphPad Prism 7.0 (San Diego, CA, USA). *p* < 0.05 was considered statistically significant.

## 3. Results

### 3.1. BRCA1-Mutant TNBC Cells Respond to Abemaciclib Inhibition

**Figure 1:**
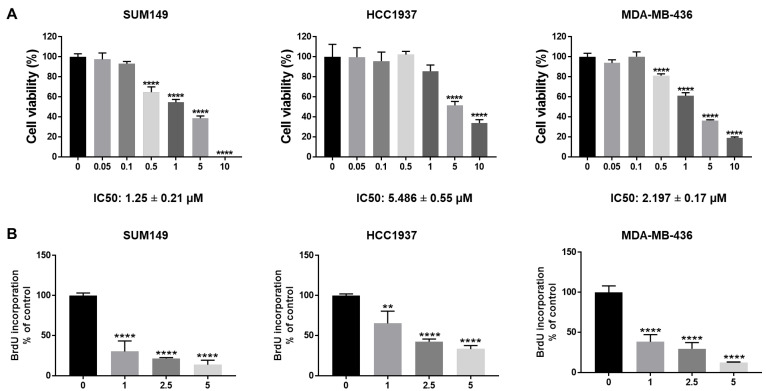
Abemaciclib inhibited *BRCA1*-mutant TNBC cell growth. SUM149, HCC1937, and MDA-MB-436 cells were treated with abemaciclib for 72 h. (**A**) Cell viability was determined by MTT assay. IC50 of abemaciclib was calculated and the IC50 ± SE values were listed at the bottom. (**B**) Cell proliferation was measured by BrdU cell proliferation assay. The drug concentrations used in the BrdU assay were selected based on the IC50 values determined in the MTT assay (**A**) to assess proliferation at biologically relevant doses. Data represents SD from three independent experiments (n = 3). ** *p* < 0.01, **** *p* < 0.0001.

To test the efficacy of abemaciclib in *BRCA1*-mutant TNBC cells, we treated SUM149, HCC1937, and MDA-MB-436 cells with different dosages of abemaciclib (0.05–10 µM). MTT assay results showed that abemaciclib inhibited cell viability in all three *BRCA1*-mutant TNBC cell lines in a dose-dependent manner (Figure 1A). The IC50 of abemaciclib in SUM149, HCC1937, and MDA-MB-436 cells is 1.25 ± 0.21 µM, 5.486 ± 0.55 µM, and 2.197 ± 0.17 µM separately (Figure 1A). In addition, we examined the effects of abemaciclib on cell proliferation of *BRCA1*-mutant TNBC cells using a BrdU cell proliferation assay. As shown in Figure 1B, abemaciclib treatment significantly reduced BrdU incorporation compared to DMSO-treated cells. Overall, these results indicate that abemaciclib can inhibit cell viability and proliferation of *BRCA1*-mutant TNBC cells.

### 3.2. Abemaciclib Induces Senescence-Associated IL-6 Secretion in BRCA1-Mutant TNBC Cells

**Figure 2:**
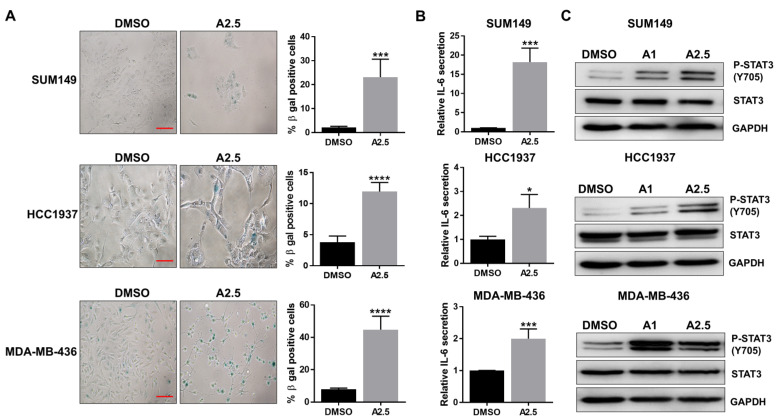
Abemaciclib induces cellular senescence and IL-6 secretion. SUM149, HCC1937, and MDA-MB-436 cells were treated with 2.5 µM abemaciclib (A2.5) for 72 h in triplicate assays. (**A**) Cells were fixed and stained for β-gal activity. Representative images shown in the left panel, scale bar is 10 µM. The percentage of β-gal positive cells are shown in the right panel. (**B**) The supernatants were collected, and the secretion of IL-6 was measured by ELISA. (**C**) SUM149, HCC1937, and MDA-MB-436 cells were treated with 1 µM abemaciclib (A1) and 2.5 µM abemaciclib (A2.5) overnight. The expression levels of P-STAT3 (Y705) and STAT3 were analyzed by western blot. GAPDH is used as a loading control. Western blot analysis was performed once due to sample limitations, * *p* < 0.05, *** *p* < 0.001, and **** *p* < 0.0001. Original western blot images can be found in the Supplementary File as Figure S1B.

Recent study has reported that inhibition of cell proliferation with CDK4/6 inhibitor can induce senescence in TNBC cells [28]. Given the inhibitory effects of abemaciclib on cell proliferation and cell cycle progression, we further investigated its impact on cellular senescence. Senescence-associated β-galactosidase (SA-β-gal) staining was performed on *BRCA1*-mutant TNBC cell lines SUM149, HCC1937, and MDA-MB-436 following treatment with abemaciclib for 72 h. The results showed a significant increase in SA-β-gal–positive cells in all three cell lines (Figure 2A). IL-6/GP130 signaling has been closely linked to cellular senescence [29]. We therefore assessed IL-6 secretion in abemaciclib-treated *BRCA1*-mutant TNBC cells using ELISA. As shown in Figure 2B, abemaciclib significantly elevated IL-6 levels in all three cell lines. We further confirmed that IL-6 levels in untreated cells were not significantly different from those in DMSO-treated cells (Figure S1A), indicating that DMSO alone does not affect IL-6 secretion in these *BRCA1*-mutant TNBC cell lines. Since STAT3 is a major downstream effector of IL-6/GP130 signaling and is known to regulate cell proliferation, survival, and drug resistance, we next examined STAT3 activation by assessing phosphorylation levels via western blot. The results demonstrated a marked increase in P-STAT3 in cells treated with 1 or 2.5 μM abemaciclib compared to the DMSO control (Figure 2C and Figure S1C). Notably, P-STAT3 levels increased with abemaciclib in a largely dose-dependent manner, with one cell line showing a modest decrease at 2.5 µM, likely reflecting cell line-specific differences in SASP-associated IL-6/STAT3 signaling. Together, these findings indicate that abemaciclib induces senescence-associated IL-6 secretion and activates STAT3 signaling in *BRCA1*-mutant TNBC cells.

### 3.3. Bazedoxifene Enhances the Sensitivity of BRCA1-Mutant TNBC Cells to Abemaciclib

**Figure 3:**
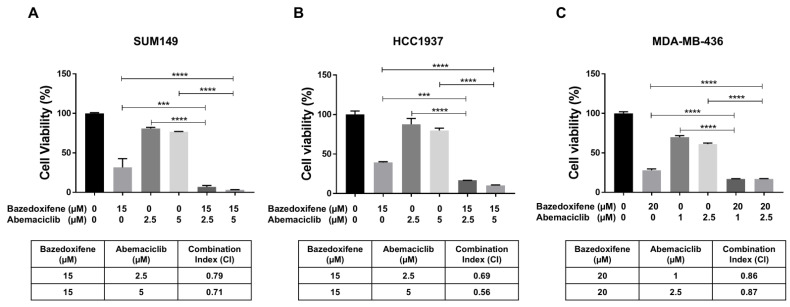
Bazedoxifene synergizes with abemaciclib in *BRCA1*-mutant TNBC cells. Cell viability was measured by MTT assay in SUM149 (**A**), HCC1937 (**B**), and MDA-MB-436 (**C**) cells after treatment with bazedoxifene alone, abemaciclib alone, and the combination for 24 h (performed in triplicate assays). The CI values were reported in the bottom panel. *** *p* < 0.001, and **** *p* < 0.0001.

To investigate the synergistic inhibitory effects of bazedoxifene and abemaciclib on *BRCA1*-mutant TNBC cells, SUM149, HCC1937, and MDA-MB-436 cells were treated with bazedoxifene, abemaciclib, or a combination of both for 72 h. Cell viability following treatment was assessed using the MTT assay and the CI was calculated. As shown in Figure 3, treatment with either bazedoxifene or abemaciclib alone reduced cell viability across all *BRCA1*-mutant TNBC cell lines. However, the combination treatment resulted in significantly greater inhibition of cell viability compared to monotherapy. Additionally, the CI values for the combination at both concentrations of abemaciclib were found to be less than 1.0 in all tested cell lines (Figure 3A, table), indicating a synergistic effect. Together, these results indicate that dual inhibition of the IL-6/STAT3 pathway and CDK4/6 synergistically suppresses the growth of *BRCA1*-mutant TNBC cells.

### 3.4. Knockdown of GP130 Increases Abemaciclib Sensitivity in BRCA1-Mutant TNBC Cells

**Figure 4:**
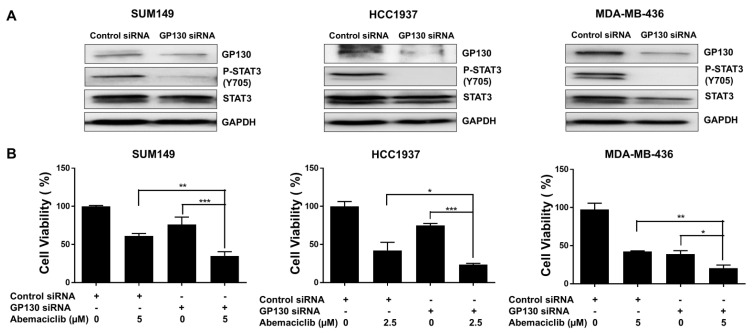
*GP130* knockdown enhances the sensitivity of abemaciclib in *BRCA1*-mutant TNBC cells. (**A**) The knockdown efficiency of *GP130* and its downstream P-STAT3 (Y705) and STAT3 were detected by western blot. GAPDH is used as a loading control. Western blot analysis was performed once due to sample limitations. (**B**) Cell viability of *GP130* knockdown alone, abemaciclib alone, and the combination treatment was measured by MTT assay (performed in triplicate assays). * *p* < 0.05, ** *p* < 0.01, and *** *p* < 0.001. Original western blot images can be found in the Supplementary File as Figure S2A.

GP130 serves as the central signal-transducing receptor in the IL-6/STAT3 pathway, initiating STAT3 activation that drives gene expression programs promoting cell survival, proliferation, and inflammation [30]. To examine the role of GP130, we silenced its expression using GP130-specific siRNA in *BRCA1*-mutant TNBC cell lines. Western blot analysis confirmed efficient knockdown, showing reduced GP130 protein levels in MDA-MB-436, HCC1937, and SUM149 cells compared to control siRNA-transfected cells (Figure 4A and Figure S2B). This reduction was accompanied by a marked decrease in P-STAT3 at Y705 (Figure 4A and Figure S2B). MTT assays revealed that GP130 knockdown significantly reduced cell viability in all three *BRCA1*-mutant TNBC cell lines compared to controls (Figure 4B). Notably, the combination of GP130 knockdown and abemaciclib treatment further decreased cell viability, suggesting that inhibiting IL-6 by silencing GP130, which decreases STAT3 phosphorylation, enhances the sensitivity of *BRCA1*-mutant TNBC cells to abemaciclib.

### 3.5. Bazedoxifene and Abemaciclib Combination Treatment Synergistically Inhibit Cell Migration in BRCA1-Mutant TNBC Cells

**Figure 5:**
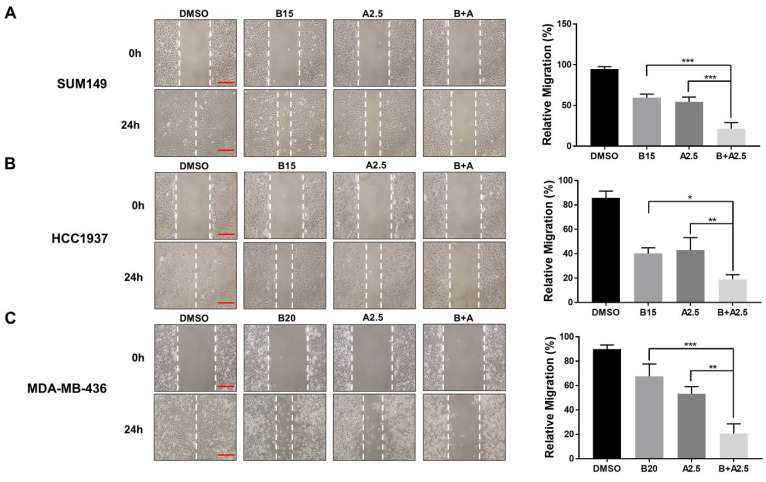
Significant inhibitory effects of bazedoxifene and abemaciclib combination on the migration of *BRCA1*-mutant TNBC cells. Wound healing assay was performed to evaluate cell migration in SUM149 (**A**), HCC1937 (**B**), and MDA-MB-436 (**C**) cells with bazedoxifene alone, abemaciclib alone, and the combination treatment for 24 h. Representative images of scratches with bazedoxifene alone (B15: 15 μM bazedoxifene; B20: 20 μM bazedoxifene), abemaciclib alone (A2.5: 2.5 μM abemaciclib), and the bazedoxifene and abemaciclib (B+A) combination treatment. The scale bar is 20 µM. The statistical analysis of cell invasion was shown in the right panel. Relative migration was quantified by measuring the wound area and statistical analysis was performed on data from triplicate assays. * *p* < 0.05, ** *p* < 0.01, and *** *p* < 0.001.

To assess the combined inhibitory effect of bazedoxifene and abemaciclib on cell migration, we performed wound healing assays using three *BRCA1*-mutant TNBC cell lines (MDA-MB-436, HCC1937, and SUM149). Representative images and quantitative analyses are shown in Figure 5. Treatment with either bazedoxifene or abemaciclib alone resulted in a moderate reduction in cell migration across all TNBC cell lines, demonstrating their individual anti-migratory effects. Notably, co-treatment with both drugs led to a significantly greater inhibition of migration compared to either single agent alone. These findings support the hypothesis that combining bazedoxifene and abemaciclib should be a more effective strategy for suppressing TNBC cell migration, potentially improving the control of tumor progression and metastasis in this aggressive breast cancer subtype.

### 3.6. Bazedoxifene and Abemaciclib Combination Treatment Synergistically Inhibit Cell Invasion in BRCA1-Mutant TNBC Cells

**Figure 6:**
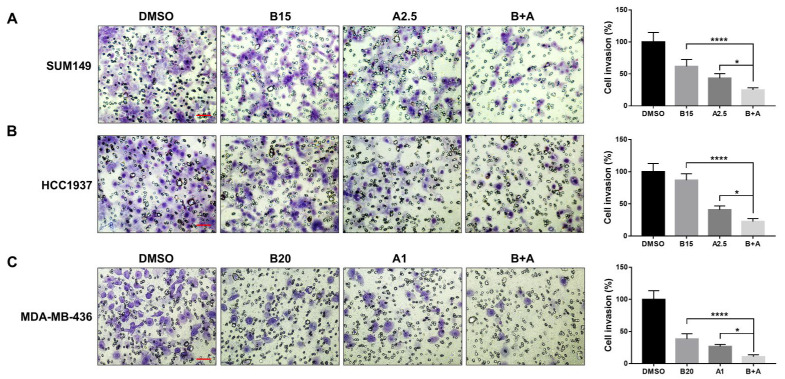
Significant inhibitory effects of bazedoxifene and abemaciclib combination on the invasion of *BRCA1*-mutant TNBC cells. Transwell invasion assay was performed in triplicate to evaluate cell invasion. Representative images of invasive cells of SUM149 (**A**), HCC1937 (**B**), and MDA-MB-436 (**C**) with bazedoxifene alone, abemaciclib alone, and the combination treatment for 24 h (B15: 15 μM bazedoxifene; B20: 20 μM bazedoxifene; A1: 1 μM abemaciclib; A2.5: 2.5 μM abemaciclib and bazedoxifene and abemaciclib (B+A) combination treatment). The scale bar is 10 µM. The statistical analysis of cell invasion was shown in the right panel. * *p* < 0.05, and **** *p* < 0.0001.

Tumor cell invasion is a hallmark of metastatic disease. To further support our migration findings, we conducted a cell invasion assay to evaluate the effects of bazedoxifene and/or abemaciclib on the invasive capacity of *BRCA1*-mutant TNBC cells. As shown in Figure 6, both bazedoxifene and abemaciclib monotherapies significantly reduced tumor cell invasion in SUM149 (A), HCC1937 (B), and MDA-MB-436 (C) cells. Notably, the combination treatment resulted in a significantly greater inhibition of invasion compared to either drug alone across all cell lines tested, highlighting the greater inhibitory efficacy of the dual drug therapy.

### 3.7. Combination Treatment of Bazedoxifene and Abemaciclib Induces Cell Apoptosis

**Figure 7:**
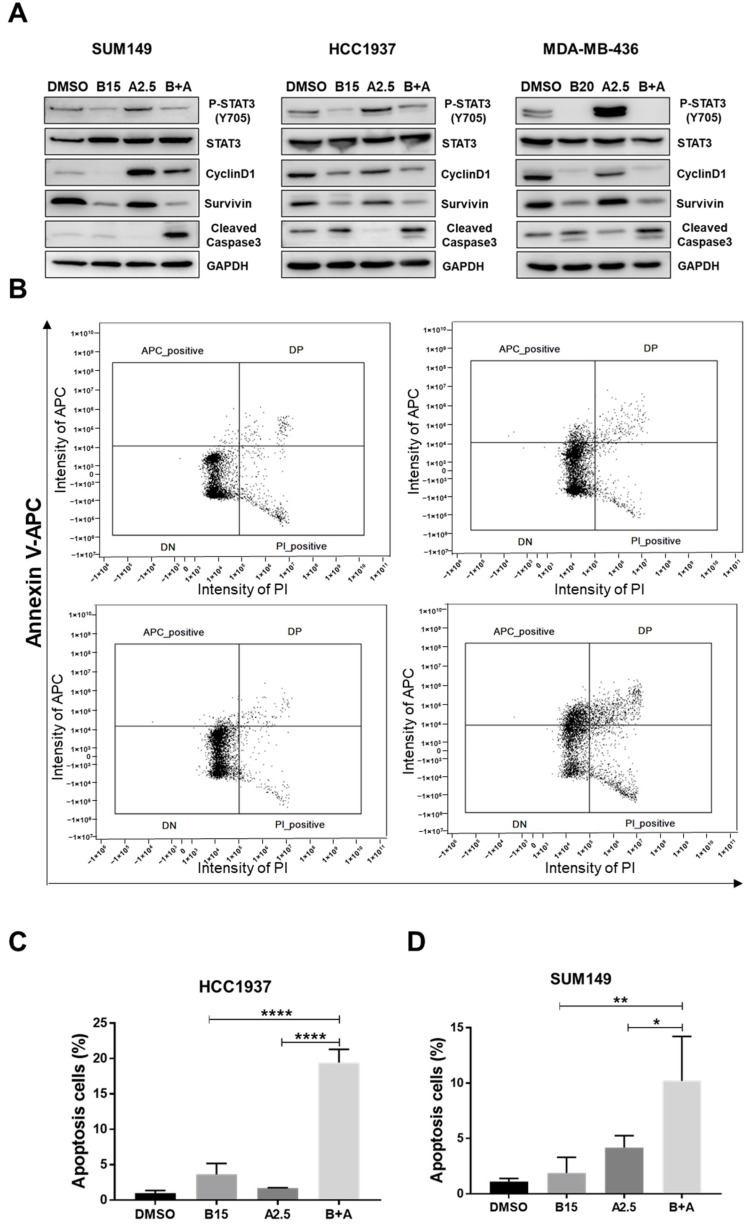
Significant greater effects of bazedoxifene and abemaciclib combination on cell apoptosis. (**A**) SUM149, HCC1937, and MDA-MB-436 cells were treated with bazedoxifene alone (B15: 15 μM bazedoxifene; B20: 20 μM bazedoxifene), abemaciclib alone (A2.5: 2.5 μM abemaciclib), and the (B+A) combination overnight. Western blot analysis was performed once due to sample limitations to assess the effects of combination on the protein levels of P-STAT3 (Y705), STAT3, cyclinD1, survivin, and cleaved caspase-3. GAPDH is used as a loading control. (**B**) HCC1937 cells were treated with 15 μM bazedoxifene (B15) alone, 2.5 μM abemaciclib (A2.5), and the combination (B+A) overnight. Annexin V-APC/PI staining was performed in triplicate and analyzed by flow cytometry to measure cell apoptosis. Apoptotic cells are labeled as APC positive and PI negative in the upper left quadrant of each diagram. Statistical analysis of cell apoptosis in HCC1937 (**C**) and SUM149 (**D**) cells. * *p* < 0.05, ** *p* < 0.01, and **** *p* < 0.0001. Original western blot images can be found in the Supplementary File as Figure S3A.

To investigate the mechanism underlying the inhibitory effects of the combined treatment with bazedoxifene and abemaciclib on *BRCA*1-mutant TNBC cells, we performed western blot analysis. As shown in Figure 7A and Figure S3B, treatment with abemaciclib alone increased phosphorylation of STAT3 compared to DMSO-treated control cells. However, when combined with bazedoxifene, phosphorylation of STAT3 and expression of its downstream targets, cyclin D1 and survivin, were significantly reduced. Additionally, the expression of cleaved caspase-3 was elevated in cells treated with the combination compared to DMSO or either single drug alone. To further confirm the induction of apoptosis, we performed Annexin V and PI staining followed by flow cytometry. As shown in Figure 7B–D, the percentage of apoptotic cells was significantly higher in the combination treatment group compared to DMSO or monotherapy in both HCC1937 and SUM149 cells. Together, these results suggest that the combination of bazedoxifene and abemaciclib induces apoptosis by inhibiting the IL-6/STAT3 signaling pathway.

### 3.8. Bazedoxifene in Combination with Abemaciclib Enhances Antitumor Activity In Vivo

**Figure 8:**
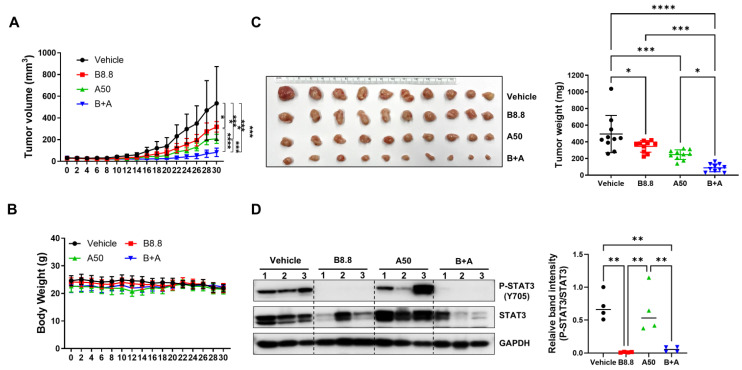
Antitumor efficacy of bazedoxifene in combination with abemaciclib in SUM149 xenograft model. Mice were injected with 5 × 10^6^ SUM149 cells in the 4th mammary fat pad bilaterally. The experimental groups were treated with vehicle control, 8.8 mg/kg bazedoxifene (B8.8), 50 mg/kg abemaciclib (A50), or the combination (B+A) one day before tumor cell inoculation. When the xenografts reached approximately 50 mm^3^, tumor volumes (**A**) and body weights (**B**) were measured every two days (n = 5 mice per group). At the end of the experiment, the xenografts were removed, and tumor weights (**C**) were measured (n = 10 tumors per group). (**D**) Western blot analysis was performed to detect the expression levels of P-STAT3 (Y705) and STAT3 in tumor lysis. GAPDH is used as a loading control. Western blot analysis was performed in three independent tumor samples. Quantification of P-STAT3 levels normalized to total STAT3, along with statistical analysis, is shown in the right panel. * *p* < 0.05, ** *p* < 0.01, *** *p* < 0.001, and **** *p* < 0.0001. Original Western blot images can be found in the Supplementary File as Figure S5.

To further evaluate the in vivo efficacy of the combination of bazedoxifene and abemaciclib, an orthotopic tumor model was performed by implanting SUM149 cells into the fourth mammary fat pad of female nude mice. Once tumors reached approximately 50 mm^3^, the mice were treated once daily by oral gavage for 30 days with either vehicle control, bazedoxifene (8.8 mg/kg), abemaciclib (50 mg/kg), or a combination of both. As shown in Figure 8A, both single-agent treatments significantly inhibited tumor growth compared to vehicle control. However, the combination therapy resulted in a markedly greater suppression of tumor growth than single drug alone and vehicle control.

Consistently, after 30 days of treatment, tumors in the combination group weighed significantly less than those in the single-agent groups (Figure 8B). Moreover, no significant changes in body weight were observed in any treatment group (Figure 8C), suggesting minimal acute toxicity. As expected, western blot analysis of tumor tissues confirmed that phosphorylated STAT3 levels were reduced in the bazedoxifene group compared to vehicle control. Notably, the combination treatment further decreased phosphorylated STAT3 levels normalized to total STAT3 compared to abemaciclib alone (Figure 8D and Figure S4B). These findings demonstrate that bazedoxifene enhances the antitumor activity of abemaciclib in vivo.

## 4. Discussion

*BRCA1* is a tumor suppressor gene essential for genomic stability, primarily through its role in repairing DNA double-strand breaks via HR [31]. Germline mutations in *BRCA1* contribute to roughly 20–25% of hereditary breast cancers and about 5–10% of all breast cancer cases. These mutations are frequently associated with high-grade, aggressive tumors and poor prognosis [32]. A significant proportion (60–80%) of *BRCA1* mutation carriers develop TNBC, an aggressive subtype characterized by the absence of hormone receptors and HER2 expression, leaving few targeted treatment options. Although PARP inhibitors have shown therapeutic benefit in *BRCA1*-mutant breast cancers, particularly in advanced stages, their effectiveness is often compromised by acquired resistance and suboptimal tumor suppression [33]. These limitations underscore the need for improved or combination treatment strategies for this challenging subset of breast cancer.

CDK4/6 are essential enzymes that govern cell cycle entry by facilitating the G1-to-S phase transition, primarily through modulation of the Rb protein [14]. Aberrant activation of the CDK4/6-Rb axis is a common feature in various malignancies, including breast cancer, promoting unchecked cellular proliferation [21,34,35]. CDK4/6 inhibitors—such as abemaciclib, palbociclib, and ribociclib—are clinically approved for use in advanced hormone receptor-positive (HR+), HER2-negative breast cancer, where they are often paired with endocrine therapy and have demonstrated marked clinical benefit [36]. In contrast, their utility in TNBC remains constrained due to the molecular diversity of TNBC and the frequent loss of functional Rb [37]. Our lab previously found that CDK4/6 inhibitors combined with bazedoxifene can synergistically inhibit *BRCA*-proficient TNBC cells in vitro [27]. However, there are currently very limited treatment options for *BRCA1*-mutant TNBC, and the therapeutic potential of CDK4/6 inhibitors in this subset remains underexplored. In the present study, we found that *BRCA1*-mutant TNBC cells are susceptible to the CDK4/6 inhibitor abemaciclib, with IC50 values of 1.25 µM for SUM149, 5.49 µM for HCC1937, and 2.20 µM for MDA-MB-436, effectively reducing cell viability and proliferation. However, treatment with abemaciclib also induces cellular senescence, accompanied by increased secretion of IL-6 (Figure 2B) and activation of STAT3 (Figure 2C). This observation is consistent with the well-established concept that senescent cells acquire a senescence-associated secretory phenotype (SASP), characterized by the release of pro-inflammatory cytokines such as IL-6 [38,39]. IL-6 is a hallmark SASP factor that reinforces the senescent state in an autocrine fashion, while also acting in a paracrine manner to remodel the tumor microenvironment [40]. Elevated IL-6 secretion from senescent cells and subsequent activation of STAT3 reinforce a pro-inflammatory microenvironment that promotes tumorigenic potential in breast cancer [29]. Upon phosphorylation, STAT3 undergoes a conformational change, dissociates from the receptor complex, forms homodimers, and translocates to the nucleus where it binds DNA and activates oncogenic transcriptional programs including genes such as *cyclin D1*, *Bcl-2*, *Bcl-xL*, *VEGF*, *VEGFR2*, and *MMPs* [41,42]. This suggests that while abemaciclib impedes tumor cell growth, it concurrently triggers a senescence-associated secretory phenotype (SASP) that may promote survival pathways and potentially contribute to therapeutic resistance.

Bazedoxifene, originally approved by the FDA for managing postmenopausal osteoporosis as part of the combination therapy Duavee with conjugated estrogens [43], has also been identified as a novel inhibitor by blocking IL-6 and GP130 interaction [44]. Recent research has demonstrated that bazedoxifene, either alone or in combination with standard chemotherapeutic agents, exhibits potent anti-tumor activity across various types of cancer including TNBC [45,46]. To address potential resistance to abemaciclib in *BRCA1*-mutant TNBC, we explored the combination of abemaciclib with bazedoxifene. We found that this combination therapy produced a synergistic effect, significantly reducing cell viability and proliferation. Similarly, knockdown of GP130, a key component of the IL-6 receptor complex, reduced phosphorylated STAT3 levels and sensitized *BRCA1*-mutant TNBC cells to abemaciclib. Since STAT3 can be activated by multiple signals, assessing IL-6 would better clarify the contribution of IL-6/GP130 signaling to abemaciclib resistance and should be addressed in future studies. Furthermore, we found that the combination of the two drugs synergistically inhibited cell migration (Figure 5) and invasion (Figure 6), and induced apoptosis (Figure 7A,D) in all three *BRCA1*-mutant TNBC cell lines. Our western blot analysis further revealed a marked increase in phosphorylated STAT3 and its downstream targets, survivin and cyclin D1, in *BRCA1*-mutant TNBC cells treated with abemaciclib alone. However, when abemaciclib was combined with bazedoxifene, levels of phosphorylated STAT3 and its downstream targets were significantly reduced. These findings suggest that targeting the IL-6/STAT3 pathway may enhance the antitumor efficacy of CDK4/6 inhibition. In vivo studies using an orthotopic TNBC mouse model demonstrated that the combination of bazedoxifene and abemaciclib significantly suppressed tumor growth (Figure 8A,B) without notable toxicity (Figure 8C). Although body weight remained stable, detailed toxicity assessments, including liver and kidney function, are needed to further confirm the safety of the combination treatment. Tumor analysis revealed decreased levels of phosphorylated STAT3 (Figure 8D). These findings underscore the translational relevance of our in vitro results and suggest that dual inhibition of CDK4/6 and IL-6/STAT3 pathways could be an effective strategy for treating *BRCA1*-mutant TNBC.

## 5. Conclusions

In summary, our study provides evidence that IL-6-mediated activation of IL-6 contributes to resistance against CDK4/6 inhibitors in *BRCA1*-mutant TNBC. Combining abemaciclib with IL-6 pathway inhibitors like bazedoxifene enhances anti-tumor efficacy, offering a potentially promising therapeutic approach. Further clinical investigations are warranted to validate these findings and explore their applicability in breast cancer patient populations.

## Data Availability

The datasets used and/or analyzed during the current study are available from the corresponding author upon request.

## Supplementary Materials

The following supporting information can be downloaded at: https://www.mdpi.com/article/10.3390/cells14201602/s1, Figure S1: (A) IL-6 secretion was measured by ELISA in supernatants from HCC1937 and MDA-MB-436 cells treated with DMSO or left untreated for 72 h. (B) Western blot shows all bands with molecular weight for Figure 2C. (C) Quantification of phosphorylated STAT3 (P-STAT3) levels normalized to total STAT3 for Figure 2C. Figure S2: (A) Western blot shows all bands with molecular weight for Figure 4A. (B) Quantification of phosphorylated STAT3 (P-STAT3) levels normalized to total STAT3 for Figure 4A. Figure S3: (A) Western blot shows all bands with molecular weight for Figure 7A. (B) Quantification of phosphorylated STAT3 (P-STAT3) levels normalized to total STAT3 for Figure7A. Figure S4: (A) Gating and border lines established using image-flow technology based on fluorescence signals from captured images. (B) Coefficient intensity plot showing residual compensation error. Figure S5: Western blot showing all bands with molecular weight for Figure 8D.

## Abbreviations

The following abbreviations are used in this manuscript:

ANOVA	One-way analysis of variance
*BRCA*	Breast cancer gene
BrdU	Bromodeoxyuridine
CDK4/6	Cyclin-dependent kinases 4 and 6
CI	Combination index
DMSO	Dimethyl sulfoxide
ELISA	Enzyme-linked immunosorbent assay
ER	Estrogen receptor
FBS	Fetal bovine serum
GAPDH	Glyceraldehyde 3-phosphate dehydrogenase
GP130	Glycoprotein 130
HER2	Human epidermal growth factor receptor 2
HR	Homologous recombination
HRP	horseradish peroxidase
IC50	Half-maximal inhibitory concentration
IL-6	Interleukin-6
LAR	Luminal androgen receptor
MAPK	Mitogen-activated protein kinase
mTOR	Mammalian target of rapamycin
MTT	3-(4,5-Dimethylthiazol-2-yl)-2,5-diphenyltetrazolium bromide
PARP	Poly (ADP-ribose) polymerase
PBS	Phosphate buffered saline
PI	propidium iodide
PI3K	Phosphoinositide 3-kinases
PKB	Protein kinase B
PR	Progesterone receptor
Rb	Retinoblastoma
P-STAT3	Phosphorylated STAT3
RPMI	Roswell Park Memorial Institute
SA-β-gal	Senescence-associated β-galactosidase
SASP	Senescence-associated secretory phenotype
siRNA	Small interfering RNA
STAT3	Signal transducer and activator of transcription 3
TNBC	Triple-negative breast cancer
