# Supplementary material for: IL-6 Blockade Enhances the Efficacy of CDK4/6 Inhibitor in BRCA1-Mutant Triple-Negative Breast Cancer Cells
Source: Cells. 2025 Oct 15;14(20):1602. doi: 10.3390/cells14201602 (PMC12564161; doi:10.3390/cells14201602)
Supplement: Supplementary file 1 [file cells-14-01602-s001.zip › cells-3801841-supplementary.pdf]

Supplemental Information

IL-6 blockade enhances the efficacy of CDK4/6 inhibitor in *BRCA1*-mutant triple-negative breast cancer cells

Figure S1

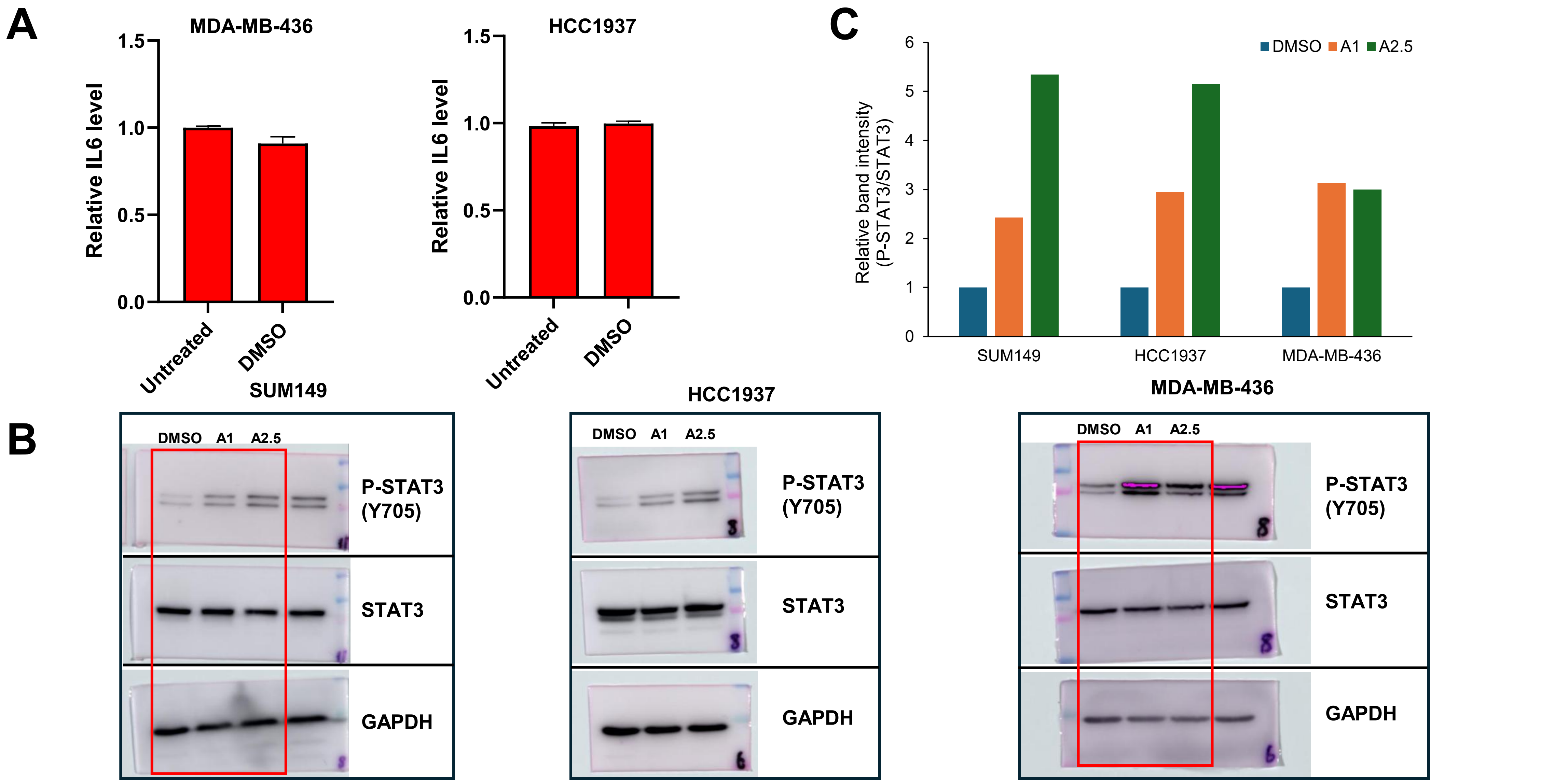

HCC1937 and MDA-MB-436 cells were treated with DMSO or left untreated for 72 h. The supernatants were collected, and IL-6 secretion was measured by ELISA in four independent replicates **(A)**. SUM149, HCC1937, and MDA-MB-436 cells were treated overnight with 1 μM abemaciclib (A1) or 2.5 μM abemaciclib (A2.5). Western blot shows all bands with molecular weights (**B**) corresponding to Figure 2C. Quantification of phosphorylated STAT3 (P-STAT3) levels normalized to total STAT3 **(C)** corresponding to Figure 2C.

Figure S2

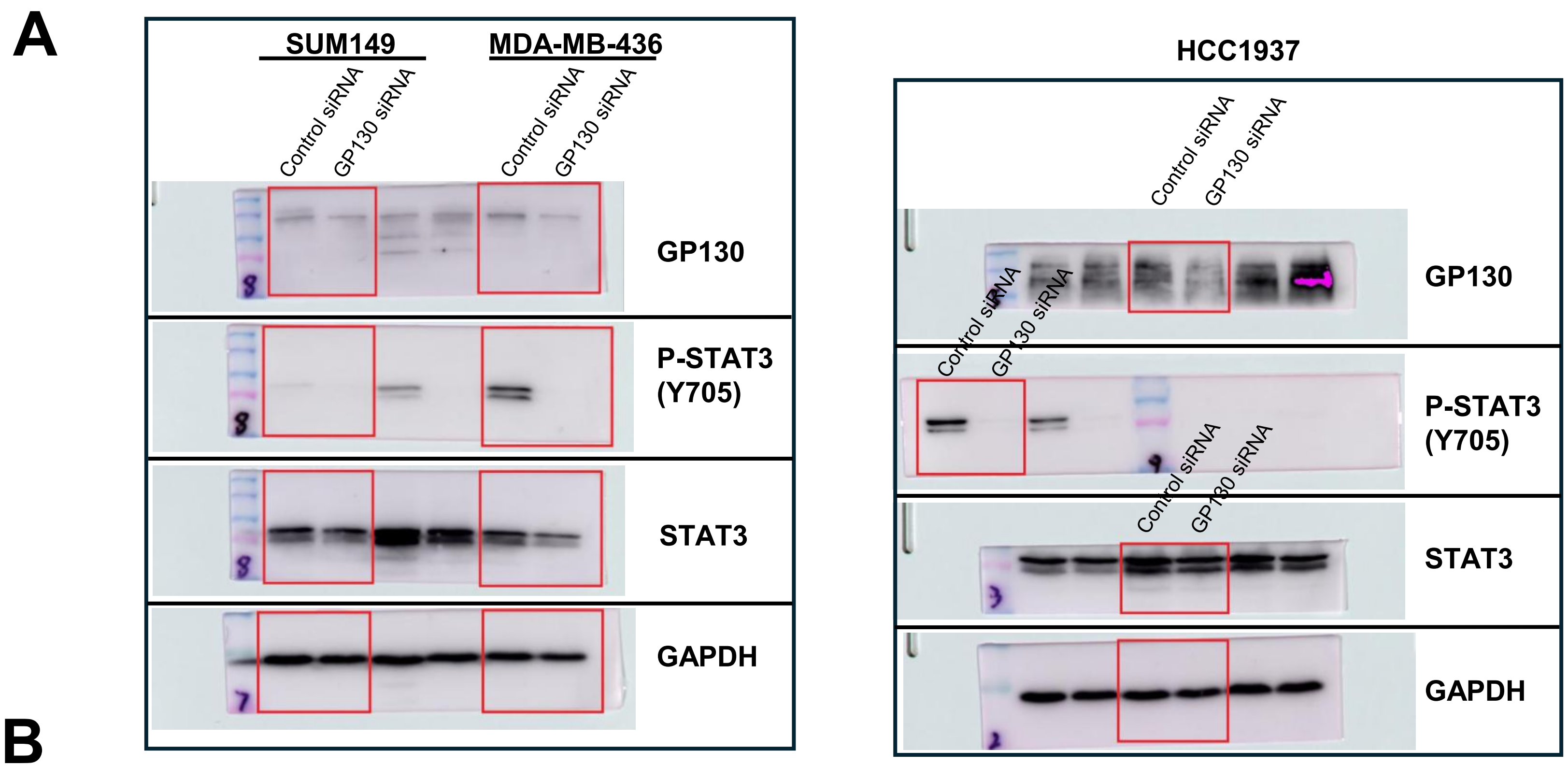

(A) Western blot shows all bands with molecular weights corresponding to Figure 4A.
(B) Quantification of STAT3, phosphorylated STAT3 (P-STAT3), and GP130 levels normalized to GAPDH, corresponding to Figure 4A.

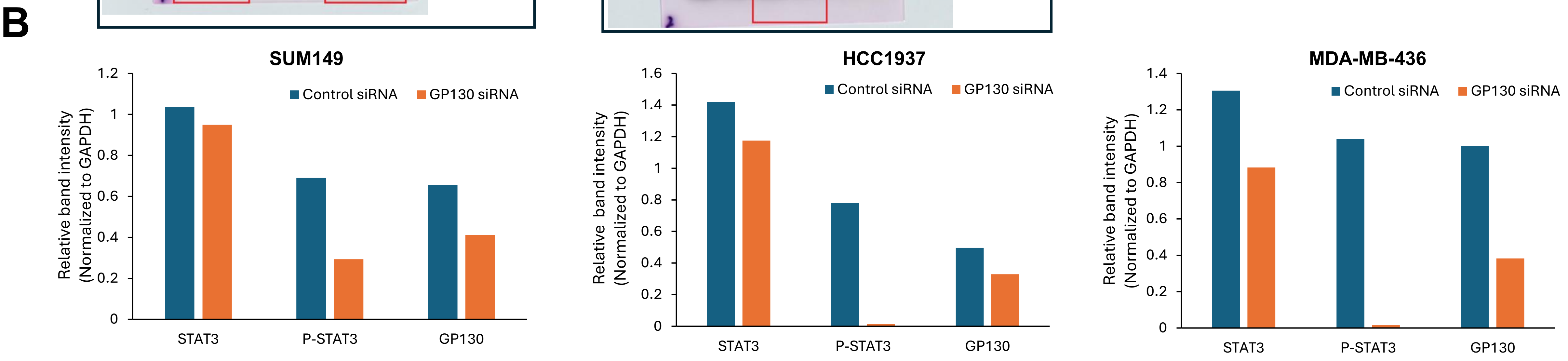

Figure S3

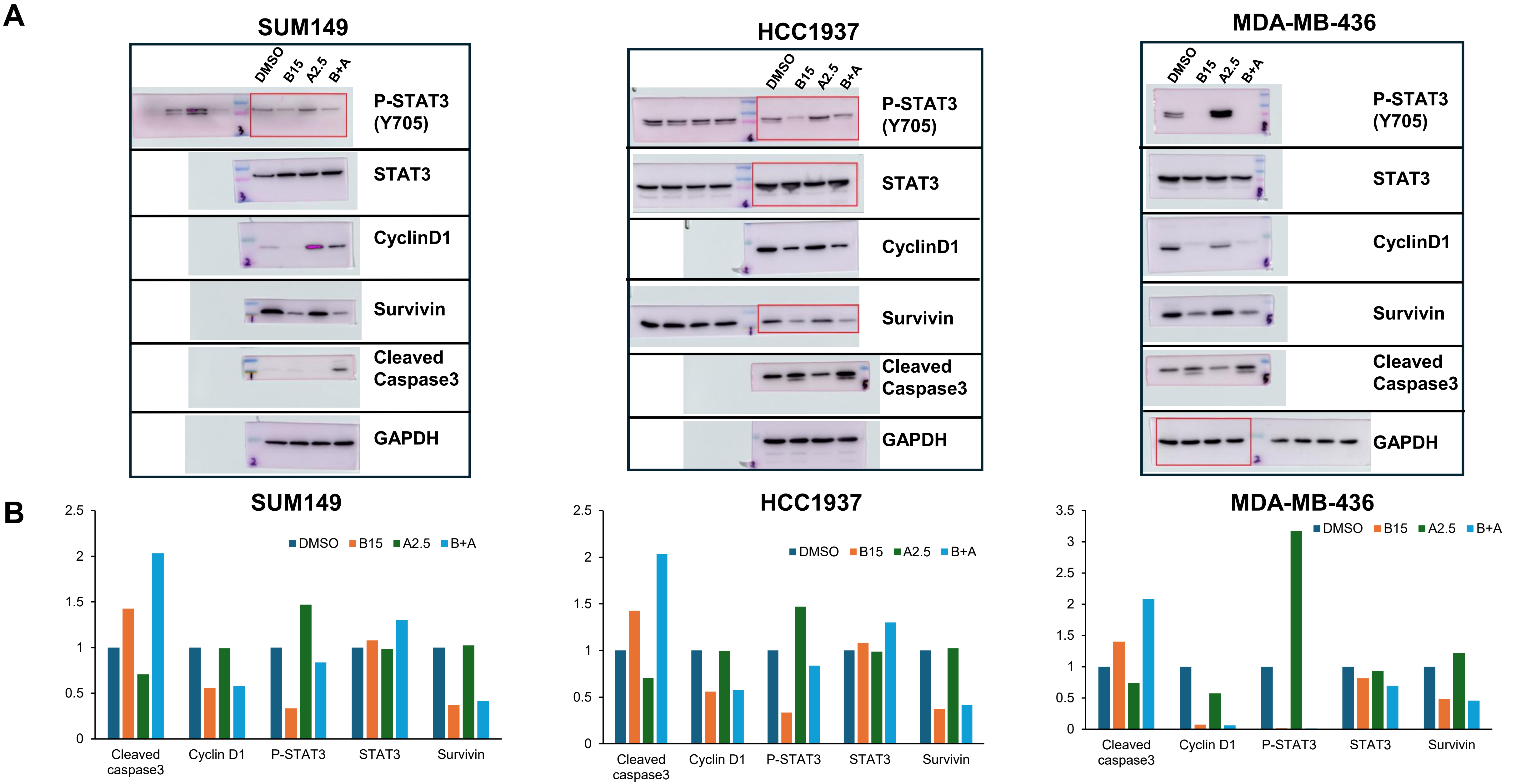

(A) Western blot shows all bands with molecular weights corresponding to Figure 7D. **(B)** Quantification of cleaved caspase3, cyclinD1, phosphorylated STAT3 (P-STAT3), STAT3 and survivin levels normalized to GAPDH, corresponding to Figure 7D.

Figure S4

A

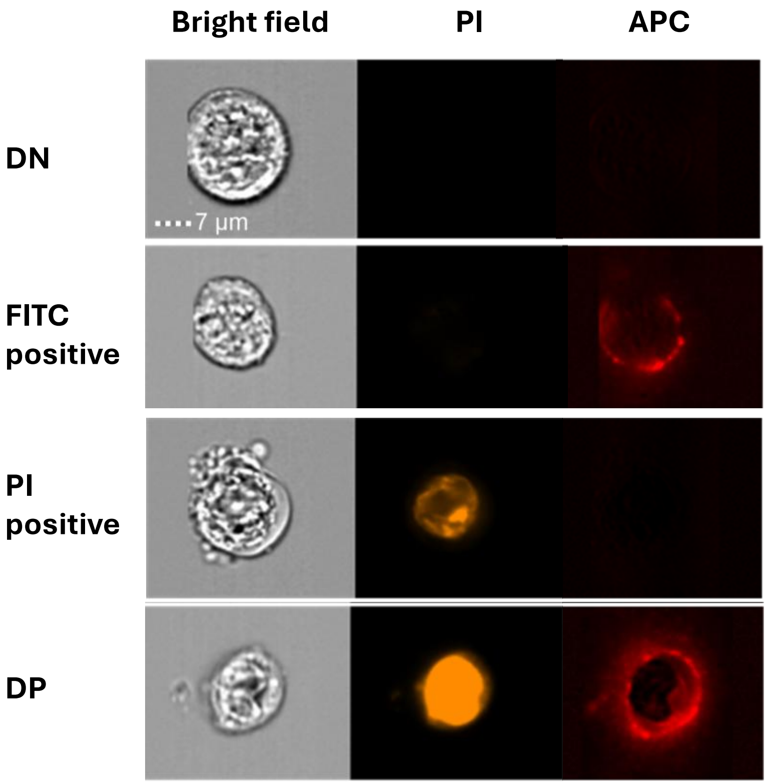

B

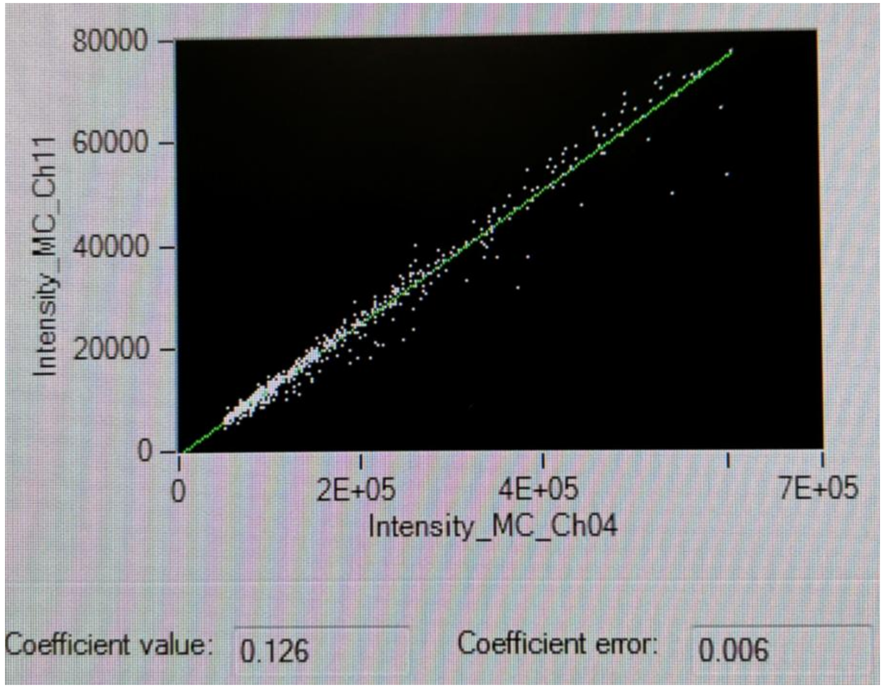

Validation of flow cytometry analysis. **(A)** Gating and border lines established using image-flow technology based on fluorescence signals from captured images. **(B)** Coefficient intensity plot showing residual compensation error <0.01, confirming accuracy of applied compensation.

Figure S5

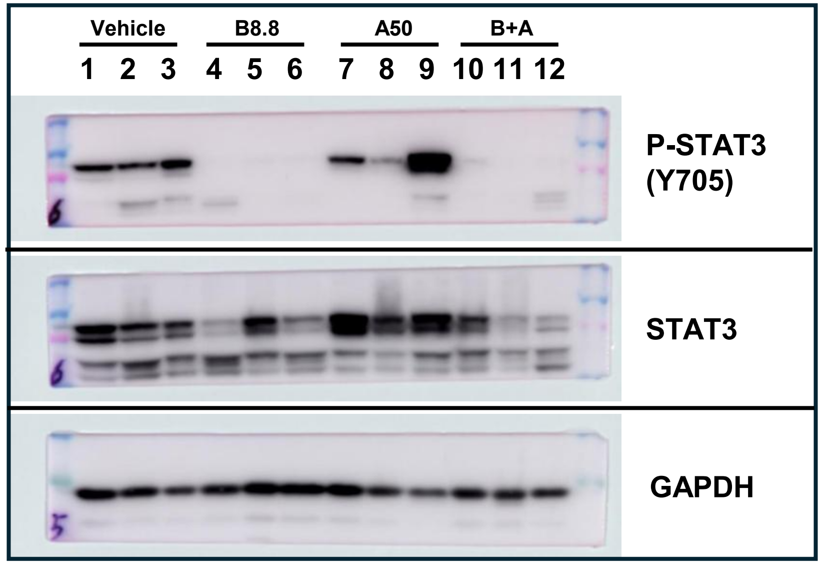

Western blot shows all bands with molecular weights corresponding to Figure 8D.
